# Supplementary material for: OpenMM 7: Rapid development of high performance algorithms for molecular dynamics
Source: PLoS Comput Biol. 2017 Jul 26;13(7):e1005659. doi: 10.1371/journal.pcbi.1005659 (PMC5549999; doi:10.1371/journal.pcbi.1005659)
Supplement: S1 Source Code — (ZIP) [file pcbi.1005659.s002.zip › OpenMM-7.0.1-Source/docs/api-c++/generated/OpenMM.OutOfPlaneSite.html]

OutOfPlaneSite — OpenMM @OPENMM\_MAJOR\_VERSION@.@OPENMM\_MINOR\_VERSION@ documentation


# OutOfPlaneSite¶

*class* `OpenMM::``OutOfPlaneSite`¶
:   This is a `VirtualSite` that computes the particle location based on three other particles’ locations. If r_1_ is the location of particle 1, r_12_ is the vector from particle 1 to particle 2, and r_13_ is the vector from particle 1 to particle 3, then the virtual site location is given by

    r_1_ + w_12_ r_12_ + w_13_ r_13_ + w_cross_ (r_12_ x r_13_ )

    The three weight factors are user-specified. This allows the virtual site location to be out of the plane of the three particles.

    Methods

    |  |  |
    | --- | --- |
    | `OutOfPlaneSite` | Create a new `OutOfPlaneSite` virtual site. |
    | `getWeight12` | Get the weight factor for the vector from particle1 to particle2. |
    | `getWeight13` | Get the weight factor for the vector from particle1 to particle3. |
    | `getWeightCross` | Get the weight factor for the cross product. |

    `OutOfPlaneSite`(int *particle1*, int *particle2*, int *particle3*, double *weight12*, double *weight13*, double *weightCross*)¶
    :   Create a new `OutOfPlaneSite` virtual site.

        |  |  |
        | --- | --- |
        | Parameters: |  |

        - **particle1** – the index of the first particle
        - **particle2** – the index of the second particle
        - **particle3** – the index of the third particle
        - **weight12** – the weight factor for the vector from particle1 to particle2
        - **weight13** – the weight factor for the vector from particle1 to particle3
        - **weightCross** – the weight factor for the cross product

    double `getWeight12`() *const*¶
    :   Get the weight factor for the vector from particle1 to particle2.

    double `getWeight13`() *const*¶
    :   Get the weight factor for the vector from particle1 to particle3.

    double `getWeightCross`() *const*¶
    :   Get the weight factor for the cross product.

High performance molecular simulation on GPUs


### Navigation

- Core Objects
- Forces
- Integrators
- Extras

©2015, Stanford University and the Authors.
|
Powered by Sphinx 1.3.1
& Alabaster 0.7.7
|
Page source
